# Biomarkers of Cellular Senescence and their Association with Frailty, Clinical Outcomes, and Survival in Multiple Myeloma

**DOI:** 10.21203/rs.3.rs-8605152/v1

**Published:** 2026-03-19

**Authors:** Nadine Abdallah, Byron Smith, Thomas White, Amanda Heeren, Tanya Wildes, Rosalyn Marar, Claire Yee, Anna Bodily, Brian Kotajarvi, Sarah Aug, Francis Buadi, Prashant Kapoor, Angela Dispenzieri, Suzanne Hayman, David Dingli, Wilson Gonsalves, Joselle Cook, Saurabh Zanwar, Moritz Binder, Yi Lin, Taxiarchis Kourelis, Morie Gertz, Rahma Warsame, S Rajkumar, Jad Sfeir, Nathan LeBrasseur, Shaji Kumar, Megan Weivoda

**Affiliations:** Mayo clinic; Mayo Clinic; Mayo Clinic; Mayo Clinic; Division of Oncology & Hematology, Department of Internal Medicine, University of Nebraska Medical Center; Mayo Clinic; Mayo Clinic; Mayo Clinic; Mayo Clinic; Mayo Clinic; Mayo Clinic; Mayo Clinic; Mayo Clinic; Mayo Clinic; Mayo Clinic; Mayo Clinic; Mayo Clinic; Mayo Clinic; Mayo Clinic; Mayo Clinic; Mayo Clinic; Mayo Clinic; Mayo Clinic; Mayo Clinic; Mayo Clinic College of Medicine; Mayo Clinic; Mayo clinic; Mayo Clinic

## Abstract

Frailty predicts adverse outcomes in multiple myeloma (MM), yet an objective frailty biomarker is lacking. Circulating senescence-associated secretory phenotype (SASP) factors correlate with frailty and adverse outcomes in chronic diseases, but their relevance in MM is unclear. In this study, we investigated the relationship between 36 plasma SASP factors, frailty, and clinical outcomes in: (1) a historical cohort using stored specimens from 59 patients with newly diagnosed MM, and (2) a prospective cohort of 36 patients initiating new MM treatment. Several SASP factors were significantly associated with frailty after adjusting for age, sex, and body mass index; GDF-15, TNF-RI, IL-6, and IL-8 showed positive correlations with both cumulative-deficit based and physical frailty indices. Additionally, significant age-adjusted associations were observed between SASP factors and treatment outcomes, including overall survival (OS), high-grade toxicity, and healthcare utilization. We developed a 5-factor SASP model that demonstrated superior predictive performance for OS compared to chronological age, R-ISS stage, and clinical frailty, and TNF-RI alone (5-year OS AUC: 0.90). These findings highlight the association between senescence biomarkers and frailty and OS, and support further investigation of SASP factors as objective frailty biomarkers in MM.

## Introduction

Multiple myeloma (MM) is a clonal plasma cell disorder that is characterized by marked heterogeneity in disease and patient characteristics, resulting in variable treatment outcomes. One factor that has emerged as a critical determinant of treatment outcomes is frailty,^1^ a multidimensional syndrome reflecting reduced physiological reserve.^2^ In MM, frailty is significantly associated with increased treatment-related toxicity and decreased overall survival (OS).^1^ Over the past decade, several tools have been developed to quantify frailty in MM,^3–8^ among which the International Myeloma Working Group (IMWG) frailty index (FI) is the most widely recognized.^1^ Despite their prognostic value, the implementation of frailty assessments in routine clinical practice remains limited, primarily due to time, resource, and workflow constraints.^9,10^ While physician-rated performance scores like the Eastern Cooperative Oncology Group (ECOG) Performance Status (PS) offer practicality, they are subjective and often discordant with the patient’s perceived physical function.^11,12^ On the other hand, objective measures like hand grip strength and gait speed, which are components of the physical frailty phenotype, are resource-consuming and confounded by disease-related symptoms. Another limitation of some existing tools is their reliance on chronological age, which can overestimate frailty in robust older adults and underestimate it in younger patients who have comorbidities and/or functional decline; we previously reported that approximately 30% of newly diagnosed MM patients less than 65 years of age in our cohort were classified as frail using an age-independent definition, underscoring that chronological age is not an appropriate surrogate for biological age.^13^ Together, these limitations have fueled interest in developing efficient and scalable biomarkers of frailty that can be readily integrated into both clinical care and research.

The hallmarks of aging, in particular cellular senescence, have emerged as attractive candidate biomarkers of frailty, as they represent the fundamental biological drivers of physiological decline.^14–17^ Cellular senescence is a state of stable growth arrest induced by cell stress. Senescent cells accumulate across tissues with aging and drive numerous aging-related pathologies, including frailty, in part through their production of proinflammatory cytokines, chemokines, growth factors, and proteases, collectively known as the senescence-associated secretory phenotype (SASP).^18^ The persistent SASP factors contribute chronic inflammation, leading to tissue dysfunction, decreased physiological reserve, and increased vulnerability to stressors.^17–20^ Recent work by Schafer et al. demonstrated that elevated levels of specific SASP factors in peripheral blood were strongly associated with clinical frailty, measured using a cumulative deficit FI, across multiple patient cohorts, and were predictive of adverse post-operative outcomes.^17^ Notably, a SASP-factor panel outperformed chronological age and the frailty index in predictive power, highlighting the potential of SASP factors as quantifiable circulating biomarkers of frailty.^17^

The utility of SASP factors as frailty and prognostic biomarkers in the context of MM remains unclear. Thus, we conducted this study to examine the association between circulating SASP factors and frailty in two separate cohorts of patients with MM, and to assess their prognostic utility significance within these cohorts.

## Methods

### Population:

To evaluate the relationship between circulating SASP factor levels and frailty, we used plasma samples from two independent patient cohorts seen at Mayo Clinic in Rochester, Minnesota: *Cohort 1* – Historical Cohort – included 59 patients with newly diagnosed MM who had stored plasma samples available in our institutional Biobank and sufficient clinical information to calculate the FI at diagnosis (see FI calculation section). Patients were diagnosed between February 19, 2010, and March 26, 2018. *Cohort 2* - Contemporary cohort – included 36 patients with newly diagnosed or relapsed/refractory MM who were scheduled to initiate a new line of therapy for MM. Peripheral blood samples were collected and frailty assessments were performed prior to treatment initiation. Patients were diagnosed between January 22, 2009, and May 31, 2024, and blood samples were collected between October 9, 2023, and June 21, 2024; the median time from diagnosis to sample collection was 2.0 months (IQR: 1–60). For the first 4 months of treatment, data was collected on treatment-related adverse events according to National Cancer Institute CTCAE v5.0,^21,22^ and on healthcare utilization (Emergency department visits/unplanned hospitalizations).

### FI calculation:

For *Cohort 1*, the FI was retrospectively calculated based on the cumulative deficit approach using 32 items abstracted from the electronic medical record, as previously described;^13^ These included 14 patient-reported items on activities of daily living (ADLs), instrumental activities of daily living (IADLs), dependence on a device for normal breathing, and exercise tolerance. The other 16 items included the body mass index (BMI) and comorbidities. Individual items were scored as 0, 0.5, or 1, with 1 indicating a deficit. The FI was defined as the sum of individual scores divided by the total number of non-missing items, and patients with >2 missing items were excluded.^13^ For *Cohort 2*, the FI was calculated using two approaches: 1) a cumulative deficit approach adapted from the CARE-FI model, integrating patient-reported geriatric and quality of life domains.^23^ To collect these data, participants completed an electronic version of the Cancer and Aging Research Group (CARG) geriatric assessment tool, which compiles validated measures of functional status, comorbidities, cognition, psychological well-being, social functioning, social support, and nutrition. In addition, patients completed the PROMIS-10 Global Health Short Form, a validated 10-item questionnaire assessing overall physical and mental health.^24,25^ A total of 40 health deficits were included in the FI, each scored as 0, 0.5, or 1, with higher scores indicating greater deficit. The overall FI was calculated as the sum of all individual deficit scores divided by the total number of non-missing items. 2) The physical frailty phenotype was determined based on 5 criteria, each scored as 0 (absent) or 1 (present): unintentional weight loss ≥10 lb. in previous year, self-reported exhaustion, weakness measured by grip strength, slow walking speed, and low physical activity.^26^ The total score ranges from 0 to 5, with 0 indicating robust, 1–2 indicating prefrail, and ≥3 indicating frail status.

### Measurement of circulating SASP factors:

The plasma concentrations of 36 SASP factors previously linked to biological aging and/or morbidity were measured.^27–29^ These included ADAMTS13, activin A, Eotaxin, Fas, GDF-15, GROα, ICAM-1, IFNγ, IL-15, IL-6, IL-7, IL-8, MCP-1, MDC, MMP-1, MMP-2, MMP-7, MMP-9, MPO, osteoactivin, osteopontin, PAI-1, PARC, PDGF-AA, PDGF-AB, PLA2G7, RAGE, RANTES, SOST, SPARC, TNF-RI, TNF-RII, TNFα, TRAIL, VEGF, and uPAR. SASP factor levels were quantified from EDTA plasma samples using commercially available multiplex magnetic bead–based immunoassays (R&D Systems) on the Luminex xMAP multianalyte profiling platform and analyzed with the MAGPIX System (Merck Millipore). Activin A was quantified using a Quantikine ELISA kit (R&D Systems), and PAI-2 was measured using an ELISA kit from Cloud-Clone Corp. All assays were performed according to the manufacturers’ instructions.

### Statistical Analysis:

Baseline characteristics were summarized using descriptive statistics. Associations between individual SASP markers and the FI (treated as a continuous variable) were assessed using Spearman and partial Spearman rank correlations, with both unadjusted and age-, sex-, and BMI–adjusted estimates reported. The adjustments for age and BMI were based on known associations between chronological age and obesity respectively, and senescent cell burden. Adjustments for sex were based on known sex differences in risk profiles for age-related diseases.^17^ Logistic regression models were used to evaluate the association between baseline SASP factor concentrations and binary outcomes, including grade ≥3 toxicity and ≥1 ED visit/unplanned hospitalization within the first 4 months of treatment (*Cohort 2* only). Univariable Cox proportional hazards models were used to evaluate the association between each biomarker and time dependent outcomes (*Cohort 1* only): Progression-free survival (PFS), TTNT (time to next treatment), and overall survival (OS). Separate bivariate models adjusting for age were also constructed. TTNT was defined as the interval from diagnosis to the initiation of the next line of therapy. Patients who had not started a new treatment at the time of last follow-up were censored at that date. PFS was defined as the time from diagnosis to documented disease progression or death from any cause, whichever occurred first. Patients alive and without evidence of progression at last follow-up were censored at that date. OS was defined as the time from diagnosis to death from any cause, with patients still alive at last follow-up censored on that date. For OS, time-dependent receiver operating characteristic (ROC) curves were generated, and the area under the curve (AUC) was calculated for each predictor at 3 and 5 years. For time-dependent models, SASP factor concentrations were log-transformed [log(x + 1)] to reduce skewness, stabilize variance, and better satisfy proportional hazards assumptions.

Parsimonious models were developed to predict OS (*cohort 1*) and FI (both cohorts) using the least absolute shrinkage and selection operator (LASSO) applied to log-transformed biomarker concentrations. LASSO was chosen to accommodate the modest sample size and address multicollinearity among predictors. The optimal penalty parameter was determined through five-fold cross-validation, and variables with non-zero coefficients at this value were retained in the final model. For the FI, a LASSO regression was fitted in *cohort 1*, with FI as the dependent variable. Model performance was assessed using R^2^, F-statistic, and residual standard error. The resulting model was externally validated in *cohort 2* by correlating LASSO-predicted frailty scores with both cumulative-deficit and physical frailty indices. For OS, a penalized Cox proportional hazards model with LASSO regularization was applied in *cohort 1*. Model discrimination was evaluated using Harrell’s concordance index (C-index) and time-dependent AUC at 3 and 5 years. An OS model was not developed for cohort 2 due to limited follow-up.

For all analyses, two-sided *p* values were reported, with *p* < 0.05 considered statistically significant. Statistical analyses were conducted using R software version 4.4.1 (R Foundation for Statistical Computing, Vienna, Austria). This study was approved by the Mayo Clinic Institutional Review Board. All participants provided written informed consent for the use of their biological samples and clinical data for research purposes.

## Results

### Patient demographic and clinical characteristics

#### Cohort 1 (newly diagnosed MM):

The median age was 60 years (IQR, 55–68), and 29% were female. Patients received proteasome inhibitor (PI)–based (27%), immunomodulatory drug (IMiD)–based (37%), or combined PI + IMiD–based (36%) induction therapy, and 41 (69%) underwent autologous stem-cell transplantation (ASCT) within one year of diagnosis. The median FI was 0.10 (IQR, 0.05–0.20).

#### Cohort 2 (Newly diagnosed and relapsed/refractory):

The median age at sample collection was 70 years (IQR: 67–74), and 16 patients (44%) were female. Twenty-three patients (64%) had newly diagnosed MM, including 3 who underwent ASCT consolidation following induction therapy; the remaining patients had relapsed or refractory disease and were initiating a new line of treatment. Seventeen patients (47%) received quadruplet-based and 19 (53%) received triplet-based regimens; half of the cohort (18 patients) received a daratumumab-containing regimen. The median FI was 0.22 (IQR, 0.13–0.37). For the physical frailty phenotype, over half of patients (53%) had a score of 0 (not frail), while 19% and 17% had scores of 1 and 2 (pre-frail), and 8% and 3% had scores 3 and 4 (frail).

### Circulating SASP factors correlate with frailty in patients with MM

#### SASP and frailty in Cohort 1:

We first evaluated the association between plasma SASP factor concentrations and the FI in *Cohort 1*. In unadjusted analyses, 10 SASP factors showed significant positive correlations with the FI: Activin A, Fas, GDF-15, IL-6, IL-8, osteoactivin, osteopontin, TNF-RI, TNF-RII, and uPAR. After adjustment for age, sex, and BMI, all associations remained statistically significant except for osteoactivin (p = 0.06). Three additional factors—GROα, RAGE, and TNF-α—became significantly correlated with frailty in adjusted models. The strongest associations were observed for TNF-RI (r = 0.501, p < 0.001), TNF-RII (r = 0.501, p < 0.001), GDF-15 (r = 0.463, p < 0.001), uPAR (r = 0.423, p = 0.001), IL-8 (r = 0.387, p = 0.003), IL-6 (r = 0.382, p = 0.004), osteopontin (r = 0.345, p = 0.009), and RAGE (r = 0.344, p = 0.009) **(Table 1)**.

#### SASP and frailty in Cohort 2:

We next examined associations between plasma SASP factor concentrations and frailty in *Cohort 2* using two distinct frailty measures: a cumulative deficit FI and the physical frailty phenotype.

#### SASP and cumulative deficit index in cohort 2:

In unadjusted analyses, factors identified in *cohort 1* including : activin A, Fas, GDF-15, IL-6, IL-8, TNF-RI, and RAGE, in addition to IFNγ, MCP-1, and MMP-7, showed significant positive correlations with the FI. After adjustment for age, sex, and BMI, all associations remained statistically significant. The strongest correlations observed were with MMP-7 (r = 0.52, p = 0.002), GDF-15 (r = 0.51, p = 0.002), IL-6 (r = 0.46, p = 0.007), IL-8 (r = 0.44, p = 0.010), IFNγ (r = 0.41, p = 0.019), Fas (r = 0.39, p = 0.027), MCP-1 (r = 0.39, p = 0.025), and TNF-RI (r = 0.39, p = 0.025) **(Table 2)**.

#### SASP and the physical frailty phenotype in cohort 2:

In unadjusted analyses, and consistent with the FI results, Fas, GDF-15, IL-6, IL-8, MMP-7, osteoactivin, and TNF-RI, along with IL-15, showed significant positive correlations with physical frailty. After adjustment for age, sex, and BMI, all associations remained statistically significant except for MMP-7 and osteoactivin. The strongest associations were observed for IL-6 (r = 0.522, p = 0.002), GDF-15 (r = 0.490, p = 0.004), TNF-RI (r = 0.437, p = 0.011), IL-15 (r = 0.412, p = 0.017), Fas (r = 0.351, p = 0.045), and IL-8 (r = 0.373, p = 0.033) **(Table 3)**.

#### A multi-factor SASP model correlates with frailty in both cohorts:

**Figure 1:**
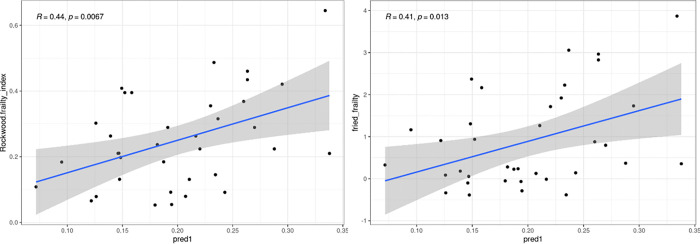
Association between SASP-based frailty model and observed frailty in *cohort 2*. Scatterplots depict the relationship between predicted frailty scores (x-axis) generated from the 3-factor SASP LASSO model developed in cohort 1 (GDF-15, TNF-RI, and uPAR) and observed frailty indices (y-axis) in *cohort 2*: (A) Cumulative deficit Frailty Index and (B) Physical Frailty Index. Blue lines represent fitted linear regressions with 95% confidence intervals (gray shading). Pearson correlation coefficients (R) and p values are shown in each panel. *Abbreviations: SASP: senescence-associated secretory phenotype.*

A LASSO regression model was fitted *in cohort 1* to predict the FI using SASP factors as predictors. The model performance was then assessed in *cohort 2*. In *cohort 1*, the model retained GDF-15, TNF-RI, and uPAR, yielding an R^2^ of 0.31 and an F-statistic of 8.37 (*p* < 0.001) with a residual standard error of 0.11. When this 3-biomarker model was applied to *cohort 2*, the predicted frailty scores demonstrated moderate to strong correlations with both the FI (r = 0.44, p = 0.007; Figure 1A) and the physical frailty phenotype (*r* = 0.41, *p* = 0.013; Figure 1B).

### Select SASP factors are associated with non-hematologic toxicity and healthcare utilization in MM

We examined the association between plasma SASP factor concentrations and treatment toxicity in *cohort 2*.

#### SASP and Grade 3–4 non-hematologic toxicity:

Five of 36 patients (14%) had grade 3–4 non-hematologic toxicity during the first 4 months of treatment. In age-adjusted analyses, higher baseline concentrations of several SASP factors were significantly associated with an increased likelihood of grade 3–4 non-hematologic toxicity. These included GDF-15 (odds ratio [OR]: 4.8, 95% CI: 1.5–29.9, p = 0.006; AUC: 0.85), Fas (OR: 3.3, 95% CI: 1.0–16.7, p = 0.04; AUC: 0.80), PAI-1 (OR: 6.3, 95% CI: 1.7–78.3, p = 0.004; AUC: 0.90), SPARC (OR: 2.7, 95% CI: 1.1–8.5, p = 0.03; AUC: 0.83), and IL-6 (OR: 3.1, 95% CI: 1.1–28.1, p = 0.03; AUC: 0.89).

#### SASP and healthcare utilization:

At least 1 ED visit and/or unplanned hospitalization(s) occurred in 15 of 36 patients (42%). In age-adjusted analyses, higher baseline concentrations of eotaxin (OR: 2.7, 95% CI: 1.2–7.4, p = 0.01; AUC: 0.76), TNF-RI (OR: 2.7, 95% CI: 1.2–8.0, p = 0.01; AUC: 0.81), TNF-RII (OR: 2.7, 95% CI: 1.2–7.3, p = 0.01; AUC: 0.79), and IFNγ (OR: 4.1, 95% CI: 1.4–21.9, p = 0.006; AUC: 0.77) were significantly associated with increased healthcare utilization. In contrast, higher concentrations of PDGF-AA (OR: 0.37, 95% CI: 0.07–0.95, p = 0.04; AUC: 0.72) and PDGF-AB (OR: 0.36,95% CI: 0.06–0.92, p = 0.03; AUC: 0.73) were significantly associated with decreased healthcare utilization

### SASP factors are associated with inferior PFS and TTNT in MM

We examined the association between plasma SASP factor concentrations and PFS and TTNT in *cohort 1*. Median follow-up was 5.0 (95% CI: 3.6–7.1) years. Elevated levels of several SASP factors were significantly associated with shorter PFS after adjusting for chronological age: Eotaxin (HR: 2.4, 95% CI: 1.1–4.9; P = 0.02), VEGF (HR: 1.9, 95% CI: 1.2–3.1; P = 0.01), RAGE (HR: 2.4, 95% CI: 1.1–5.2; P = 0.04), TNF-RII (HR = 4.4, 95% CI: 1.9–10.3; P < 0.001), GDF-15 (HR: 1.8, 95% CI 1.1–2.9; P = 0.02), TNF-RI (HR: 3.5, 95% CI: 1.6–7.7; P = 0.002), uPAR (HR: 3.7, 95% CI: 1.7–8.4; P = 0.002), Activin A (HR: 2.1, 95% CI: 1.1–3.7; P = 0.02), and IL-8 (HR: 2.7, 95% CI: 1.3–5.7; P = 0.008). Among these factors, RAGE (HR: 2.4, 95% CI: 1.0–5.8, P=0.046), TNF-RII (HR: 16.7, 95%CI: 4.9–64.5, P<0.001), TNF-R1 (HR: 10.0, 95% CI: 3.3–30.9, P<0.001), and uPAR (HR: 5.7, 95% CI: 1.7–21.4, P=0.007) were significant associated with shorter TTNT.

### SASP factors are associated with inferior OS in MM

**Figure 2:**
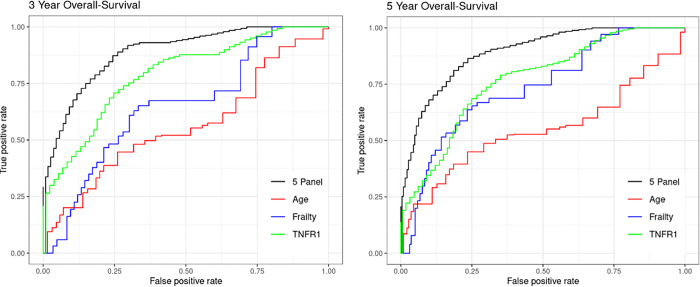
Predictive performance of a 5-factor SASP models for overall survival. Receiver operating characteristic (ROC) curves showing the performance of different models—SASP 5-factor panel (black), chronological age (red), frailty score (blue), and TNF-RII (green)—for predicting (A) 3-year and (B) 5-year overall survival in patients with newly diagnosed multiple myeloma (*Cohort 1*). The SASP 5-factor panel includes IL-8, Osteopontin, SOST, TNF-RI, and uPAR.

Finally, we examined the association between plasma SASP factor concentrations and OS in *cohort 1*. In Cox models using log-transformed biomarker concentrations, several SASP factors were significantly associated with inferior OS after adjusting for age, including activin A, ICAM-1, GDF-15, IL-8, MMP-7, osteopontin, RAGE, SOST, TNF-RI, TNF-RII, uPAR, and VEGF. The strongest predictors of OS were TNF-RI (HR: 10.5, C-index = 0.813, p < 0.001), TNF-RII (HR: 11.9, C-index = 0.789, p < 0.001), uPAR (HR: 7.8, C-index = 0.781, p < 0.001), IL-8 (HR: 5.3, C-index = 0.744, p < 0.001), and GDF-15 (HR: 2.9, C-index = 0.786, p < 0.001) **(Table 4)**. A LASSO-Cox model was developed to predict OS using SASP factors as predictors. The model for OS retained IL-8, osteopontin, SOST, TNF-RI, and uPAR. This 5-factor model demonstrated superior predictive performance for OS compared with chronological age, R-ISS stage, the FI, and TNF-RI alone. The 5-year AUC values were chronological age: 0.57, R-ISS stage III (vs I/II): 0.61, FI: 0.75, TNF-RI: 0.78, and the SASP panel: 0.90 (Figure 2B). The corresponding AUC values for 3-year OS were 0.58, 0.58, 0.67, 0.80, and 0.89, respectively (Figure 2A).

## Discussion

The SASP is increasingly recognized as a key mediator of aging-related conditions, including frailty, and is linked to adverse clinical outcomes across diverse populations.^17–20,30–33^ Although some differences were observed, GDF-15, TNF-RI, IL-6, and IL-8 consistently correlated with frailty across both patient cohorts, as measured by both clinical and phenotypic frailty indices. These findings partially align with Schafer et al., who reported associations between TNF-R1, GDF-15, and osteopontin with frailty index scores across three independent cohorts (older adults undergoing aortic stenosis surgery, older adults undergoing ovarian cancer surgery, and community biobank participants), while activin A and IL-6 were associated with frailty primarily in nonsurgical populations.^17^

Our analysis also revealed that several SASP factors were associated with reduced PFS and OS in newly diagnosed MM patients, suggesting that increased SASP levels may reflect an interplay between disease burden and biological aging. We hypothesize that MM-related inflammation may accelerate cellular senescence and physiological decline, manifesting as disease-related frailty in some patients. This is supported by reports showing that frailty status improvements in a subset of patients following effective MM therapy.^34,35^

We have previously demonstrated that cellular senescence influences disease evolution from monoclonal gammopathy of undetermined significance to symptomatic MM.^36^ Moreover, individual SASP factors may exert distinct effects at the intersection of aging and disease progression. For example, in our previous study, GDF-15 and TNF-R1 were enriched in patients with stable smoldering MM, suggesting these markers may be independent of MM pathogenesis. In active MM, Corre et al. reported GDF-15 overexpression by bone marrow mesenchymal stromal cells enhanced stromal support, promoting aggressive disease features, treatment resistance, and inferior clinical outcomes.^37^ Consistent with this, we found that elevated GDF-15 in MM was significantly associated with shorter PFS and OS. Other SASP factors, including eotaxin, SOST, and ICAM-1, were linked to clinical outcomes but not frailty.

Notably, only GDF-15, Fas, PAI-1, SPARC, and IL-6 were significantly associated with high grade non-hematologic toxicity after adjusting for age. However, the overall incidence of high-grade toxicity in *cohort 2* was low, possibly reflecting improved tolerability of contemporary treatment regimens and/or the limited sensitivity of CTCAE grading in capturing patient symptoms. Additionally, only TNF-R1, TNF-RII, eotaxin, and IFN-γ were significantly associated with ED visits/unplanned hospitalizations, likely due to disease-related complications (e.g., bone pain, pathological fracture) and/or patient-specific factors comorbidities.

The consistent associations of GDF-15, TNF-R1, and IL-6 with both frailty and adverse outcomes across oncologic and non-oncologic settings suggest that these factors contribute to frailty through mechanisms extending beyond MM biology. Furthermore, their superior prognostic performance compared to disease stage and chronological age further indicates that these associations with clinical vulnerability are not solely driven by disease burden. Consistent with the findings of Schafer et al., our findings support the use of a multi-marker SASP panel for improved prognostic accuracy compared to a single biomarker.^17^

Beyond their prognostic potential, SASP factors could serve as modifiable therapeutic targets through both pharmacologic and non-pharmacologic strategies. These include senolytics, which selectively induce apoptosis in senescent cells, and senomorphics, which modulate senescent cell activity without causing cell death. The senolytic combination of dasatinib plus quercetin (D+Q), has been shown to lower circulating SASP factors and reduce senescence-associated dysfunction in preclinical^38,39^ and early clinical studies.^40^ JAK inhibition also suppressed SASP production and alleviated age-related frailty in a preclinical study.^41^ Similarly, NF-κB pathway inhibition was shown to mitigate chemotherapy-induced cachexia and sarcopenia in a preclinical study by reducing senescence-mediated signaling including SASP secretion.^42^ SASP modulation has also been achieved with non-pharmacologic interventions: in a study by Englund et al., 12 week structured exercise interventions were associated with significant reductions in SASP factor levels and improvement in physical function in older adults, with baseline SASP levels predicting functional improvements.^28^

Our study has several limitations including modest sample size, single institution design, and predominantly white population, which may limit the generalizability of our findings. Due to small sample size, our analyses were not sufficiently powered to assess subgroup effects or to adjust for all potential confounders. Additionally, the relatively short follow-up period in *cohort 2* precluded evaluation of survival outcomes in this group. We also acknowledge that while senescent cells are a plausible source of the cytokines, chemokines, tissue remodeling, growth factor, and other proteins measured, they are not the only source. Despite these limitations, to our knowledge, this study represents the first systematic evaluation of circulating SASP factors as correlates of frailty in MM. The replication of key findings across two independent cohorts supports the internal consistency and robustness of our results. Notably, although cohort 2 was small, it was prospectively designed and included comprehensive and prospectively collected data on toxicity and healthcare utilization. Our observations and reported associations need to be validated in larger studies with more diverse populations. Beyond SASP factors, other molecular markers of biological aging—such as p16ÎNK4â expression and epigenetic clocks—may also serve as objective measures of frailty in patients with MM, as suggested by recent work from Rosko et al., and warrant further investigation.^14^

## Conclusion

In conclusion, we found that specific SASP proteins are associated with frailty in patients with MM and are predictive of inferior OS. These findings highlight the potential for SASP factors as objective biomarkers of physiological reserve, complementing traditional clinical assessments and warrant further validation in larger prospective studies.

## Supplementary Material

Supplementary Files

This is a list of supplementary files associated with this preprint. Click to download.

• Tables.docx

Tables are available in the Supplementary Files section.

## Data Availability

The datasets analyzed for this study are provided upon reasonable request from the corresponding author.
